# The impact of Smartphone addiction on depression symptoms among college students: the mediating role of bedtime procrastination and the moderating effect of psychological resilience

**DOI:** 10.3389/fpsyg.2026.1784047

**Published:** 2026-04-29

**Authors:** Zeng Zhou, Shiyuan Zhang, Zhong Li, Wei Feng, Fulan Zhang, Aolun Wang, Chuqi Yan

**Affiliations:** 1 Department of Physical Education, Central South University, Changsha, Hunan, China; 2 Melbourne Graduate School of Education, University of Melbourne, Melbourne, VIC, Australia; 3 School of Sports Science, Jishou University, Jishou, Hunan, China

**Keywords:** bedtime procrastination, college students, depression symptoms, psychological resilience, Smartphone addiction

## Abstract

**Objective:**

To investigate the influence of Smartphone addiction on depression symptoms among college students, examine the mediating role of bedtime procrastination, and explore the moderating effect of psychological resilience.

**Methods:**

Between October and December 2024, a stratified random cluster sampling method was employed to select 1,610 college students from four universities as research subjects. The Mobile Phone Addiction Index (MPAI), Bedtime Procrastination Scale (BPS), Patient Health Questionnaire-9 (PHQ-9), and Connor-Davidson Resilience Scale (CD-RISC-10) were used for data collection. Correlation analysis, mediation effect analysis, and moderated mediation model testing were conducted to analyze the relationships among variables.

**Results:**

(1) Depression symptoms were significantly positively correlated with Smartphone addiction (*r* = 0.456, *p* < 0.01) and bedtime procrastination (*r* = 0.308, *p* < 0.01), and significantly negatively correlated with psychological resilience (*r* = −0.331, *p* < 0.01). (2) Bedtime procrastination partially mediated the relationship between Smartphone addiction and depression symptoms, with an indirect effect of 0.284 (95% CI: 0.226~0.347), accounting for 19.08% of the total effect. (3) Psychological resilience significantly moderated the direct pathway between Smartphone addiction and depression symptoms (β=-0.017, *p* < 0.01), with individuals higher in psychological resilience demonstrating a weaker positive correlation between Smartphone addiction and depression symptoms. Simultaneously, psychological resilience significantly moderated the relationship between bedtime procrastination and depression levels (β = −0.004, *p* < 0.01), with a weaker positive correlation between bedtime procrastination and depression symptoms observed in individuals with higher levels of psychological resilience.

**Conclusion:**

A moderated mediation model exists between Smartphone addiction and depression symptoms among college students, with bedtime procrastination serving as a partial mediator. Therefore, in preventing and intervening in depression symptoms among college students, attention should be paid to reducing excessive mobile phone use, improving sleep habits, and cultivating psychological resilience to enhance their ability to cope with challenges and stress, thereby effectively reducing the risk of depression symptoms.

## 1 Introduction

With the ubiquity of mobile internet technology, smartphones have become deeply embedded in the daily lives of college students, serving as essential tools for social interaction, information acquisition, and entertainment. However, the omnipresence of these devices has given rise to a maladaptive psychological phenomenon known as Smartphone addiction. Conceptually, Smartphone addiction is defined as a behavioral addiction characterized by compulsive usage, withdrawal symptoms (e.g., anxiety when separated from the phone), tolerance (needing increased usage to achieve satisfaction), and functional impairment in daily life (Monday et al., 2021; Sohn et al., 2019). It should be noted that “Smartphone addiction” remains a controversial concept in the academic community. Some scholars argue that its core features are analogous to substance addiction, classifying it as a behavioral addiction. In contrast, other researchers prefer terms such as “problematic smartphone use” to emphasize dysfunctional usage patterns rather than a pathological state of addiction (Panova and Lleras, 2016; van Deursen et al., 2015). Acknowledging this debate, our study employs the term “Smartphone addiction” primarily to maintain consistency with the conceptual framework of our measurement tool, the Mobile Phone Addiction Index (MPAI). We operationally define it as a state in which excessive smartphone use leads to functional impairment in an individual's daily life. Recent epidemiological data indicates a worrying trend; a 2024 cross-temporal meta-analysis revealed that the prevalence of Smartphone addiction among college students has been steadily climbing over the past decade, exacerbated by the digitalization of education post-pandemic (Billieux et al., 2015). This upward trend has been consistently observed across global cultural contexts, with research in Brazil documenting a 62.6% prevalence of Smartphone addiction among nursing students during the COVID-19 pandemic, alongside a significant association between Smartphone addiction and moderate to severe depressive and anxiety symptoms (dos Santos and de Oliveira, 2022). College students, situated in a critical developmental transition from adolescence to adulthood, face heightened academic pressure and social challenges (Duan et al., 2020). In this context, the smartphone often transforms from a tool into a “digital pacifier,” leading to excessive dependence. While existing literature has extensively explored the antecedents of addiction, such as personality traits (Kim et al., 2018), there is an urgent need to elucidate the cascading consequences of this behavior on mental health through a coherent theoretical lens.

Depressive symptoms are among the most common emotional disorders in adolescents and are recognized as a major public mental health concern worldwide (McGrath et al., 2023). Based on the dimensional model of depressive symptoms, they can be classified into three stages: no depression, subthreshold depression, and clinical depression (Costello et al., 2003). Clinical depression is typically defined as a mood disorder characterized by a significant and persistent low mood resulting from various causes (Ayuso-Mateos et al., 2010). Subthreshold depression refers to a psychological subclinical state in which individuals exhibit certain depressive symptoms but do not meet the diagnostic criteria for clinical depression. It represents a transitional condition between mental health and clinical depression (Hamilton et al., 2012).

To understand the link between Smartphone addiction and these depressive symptoms, this study adopts the Conservation of Resources (COR) (Hobfoll, 1989) theory as its overarching framework. COR theory posits that individuals strive to obtain, retain, and protect resources (e.g., time, energy, social support, and self-esteem), and psychological stress occurs when these resources are threatened or lost (Hobfoll et al., 2018). In the context of the present study, severe Smartphone addiction is viewed as a behavior that triggers a chronic “resource loss spiral.” The compulsive cognitive engagement and emotional investment in virtual environments excessively drain an individual's limited cognitive and temporal resources required for real-world coping (Elhai et al., 2017). This resource depletion impedes their ability to cope with academic and social demands. Furthermore, the social isolation often accompanying excessive screen time deprives individuals of social support resources, ultimately rendering them more vulnerable to depressive symptoms. While empirical studies have confirmed a positive correlation between Smartphone addiction and depression (Lapierre et al., 2019; Yang et al., 2020), the specific mechanisms—how resource depletion translates into pathology—require further validation. Thus, we propose Hypothesis 1: Smartphone addiction is positively correlated with depressive symptoms.

Previous research suggests that the path from addiction to depression is not solely direct but mediated by behavioral factors, most notably Bedtime Procrastination. Defined by Kroese et al., bedtime procrastination is the volitional delay of going to bed, without external reasons, resulting in insufficient sleep (Kroese et al., 2014). Within the COR framework, sleep is the primary physiological mechanism for resource restoration. However, Smartphone addiction disrupts this restoration process. Due to the “flow” experience and immediate gratification algorithms of mobile apps, addicted users often fail to disengage psychologically at night, leading to sleep displacement (Zhang and Wu, 2020). This behavioral pattern creates a “double jeopardy” for resources: the addiction consumes energy during the day, and bedtime procrastination blocks the recovery of energy at night (Baglioni et al., 2011). The resulting sleep deprivation disrupts circadian rhythms and neuroendocrine functioning (Chung et al., 2020), rendering the individual emotionally vulnerable and significantly increasing the risk of depression (Geng et al., 2021). Thus, bedtime procrastination may act as a critical bridge where behavioral dysregulation transforms into emotional disorder. We propose Hypothesis 2: Bedtime procrastination mediates the relationship between Smartphone addiction and depressive symptoms.

While the “Addiction → Procrastination → Depression” pathway outlines a general risk mechanism, not all addicted students develop depression, highlighting the role of individual differences. Psychological Resilience is defined as the dynamic process of adapting well in the face of adversity, trauma, tragedy, or significant sources of stress (Southwick et al., 2014). This trait can be effectively measured using standardized scales such as the CD-RISC (Connor and Davidson, 2003). According to the buffer hypothesis of the COR theory, personal resource reserves (such as resilience) can buffer the impact of resource loss on mental health (Tugade and Fredrickson, 2004). First, resilience may moderate the direct impact of addiction on depression. High-resilience individuals typically possess superior emotional regulation capabilities and cognitive flexibility. Even when struggling with smartphone usage control, they can mobilize internal protective resources to reframe negative experiences and maintain self-efficacy, thereby preventing the resource loss of addiction from directly escalating into depressive despair (Nolen-Hoeksema, 1991). Conversely, low-resilience individuals, lacking these protective buffers, are more likely to succumb to the stress of addiction. Thus, we propose Hypothesis 3: Psychological resilience moderates the direct relationship between Smartphone addiction and depressive symptoms; specifically, this association is weaker for individuals with high psychological resilience.

Crucially, resilience may also moderate the second stage of the mediation process (Bedtime Procrastination → Depressive Symptoms). This path represents the transition from physiological depletion (lack of sleep) to psychological pathology (depression). Sleep deprivation impairs the prefrontal cortex's control over the amygdala, usually leading to negative emotional reactivity (Goldstein and Walker, 2014). However, individuals with high psychological resilience possess stronger “top-down” cognitive control resources. Even in a state of fatigue caused by bedtime procrastination, they can utilize positive coping strategies (e.g., cognitive reappraisal) to manage the negative affect arising from sleep loss, cutting off the path to depression (Cohen and Wills, 1985; Li et al., 2021). In contrast, for individuals with low resilience, the resource depletion from sleep loss is more likely to trigger catastrophic thinking and emotional collapse (Hu et al., 2015), confirming the onset of depression. Therefore, resilience acts as a “safety valve” at the final stage of the risk model (Hershner and Chervin, 2014). We propose Hypothesis 4: Psychological resilience moderates the relationship between bedtime procrastination and depressive symptoms; specifically, this association is weaker for individuals with high psychological resilience.

**Figure 1:**
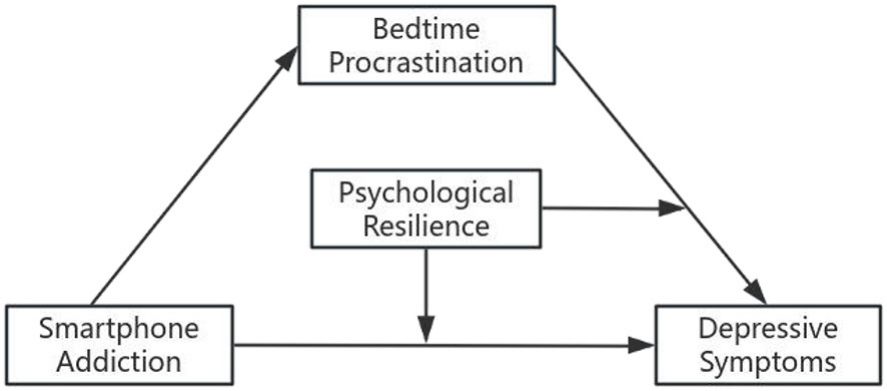
This figures shows a moderated mediation model.

In summary, despite isolated studies linking these variables, there is a lack of an integrated model examining how Smartphone addiction translates into depression through sleep displacement and under what conditions this process is buffered. This study aims to fill this gap by constructing a moderated mediation model (Figure 1) based on COR theory (Liu et al., 2017). By clarifying these mechanisms, we aim to provide theoretical evidence for developing resilience-based interventions to mitigate the depression risk associated with digital overuse in college students.

## 2 Subjects and methods

### 2.1 Research participants

This study was conducted from October 2024 to December 2024 using stratified random cluster sampling to select participants. First, four universities were selected as research sites: Central South University and Changsha University of Science and Technology in Changsha, Hunan Province, and Guizhou University and Guizhou Normal University in Guiyang, Guizhou Province. Next, stratification was performed according to departments and grade levels (freshman through senior) within each university. Finally, using a random number table, four administrative classes were selected from each department and grade level across the universities, totaling 64 classes with 1,610 students participating in the survey. After rigorous screening, 1,422 valid questionnaires were obtained, yielding an effective response rate of 88.32%. As detailed in Table 1, the sample included 901 males (63.4%) and 521 females (36.6%). Participants were evenly distributed across academic years: 360 freshmen (25.3%), 357 sophomores (25.1%), 348 juniors (24.5%), and 349 seniors (24.5%). The academic majors were also balanced, with 702 students from liberal arts (49.4%) and 720 from sciences (50.6%). With regard to family background, 888 students (62.4%) were not only children, and 1,008 (70.9%) were from urban residences. Table 1 also presents the results of *t*-tests and one-way ANOVAs, which examined differences in the primary study variables across these demographic groups.

**Table 1:** Analysis of differences in study variables by demographic characteristics (M ± SD).

Variable	Group	N	Depression symptoms	Mobile Phone Addiction	Psychological resilience	Bedtime procrastination
Gender	Male	901	3.80 ± 4.292	35.18 ± 13.926	28.75 ± 7.840	26.12 ± 5.823
	Female	521	4.12 ± 4.251	36.06 ± 14.086	28.31 ± 8.084	26.03 ± 6.638
	T		1.855	1.304	1.049	0.075
	P		0.173	0.254	0.036	0.784
Grade	Freshman	360	3.37 ± 3.577	31.28 ± 11.834	28.88 ± 7.572	25.62 ± 4.888
	Sophomore	357	3.49 ± 4.148	31.48 ± 12.359	29.97 ± 8.017	23.69 ± 6.699
	Junior	348	3.88 ± 3.399	38.94 ± 11.444	28.15 ± 7.129	27.48 ± 5.629
	Senior	349	4.97 ± 5.541	40.65 ± 11.45	27.37 ± 8.748	27.76 ± 6.484
	F		10.364	47.853	6.957	35.431
	P		0.001	0.001	0.001	0.001
Ethnicity	Han	1266	3.93 ± 4.345	35.52 ± 13.971	28.70 ± 7.929	26.16 ± 6.008
	Tujia	24	4.42 ± 3.283	40.29 ± 15.904	27.92 ± 8.382	23.67 ± 8.223
	Miao	17	4.53 ± 3.625	33.12 ± 15.447	27.76 ± 7.766	25.18 ± 6.858
	Others	115	3.55 ± 3.796	34.48 ± 13.418	27.66 ± 7.930	25.91 ± 6.810
	F		0.506	1.568	0.722	1.459
	P		0.687	0.195	0.539	0.224
Major	Liberal arts	702	4.11 ± 4.380	35.98 ± 14.319	28.14 ± 9.082	25.29 ± 6.795
	Science	720	3.86 ± 4.251	35.37 ± 13.899	28.71 ± 7.588	26.30 ± 5.926
	T		0.796	0.448	1.250	6.431
	P		0.372	0.504	0.264	0.011
Only child	Yes	534	3.50 ± 4.138	34.78 ± 14.379	29.16 ± 7.841	25.52 ± 6.559
	No	888	4.17 ± 4.343	35.93 ± 13.736	28.25 ± 7.968	26.42 ± 5.837
	T		8.133	2.255	4.402	7.303
	P		0.004	0.133	0.036	0.007
Family residence	Rural	414	4.49 ± 4.493	37.53 ± 14.413	23.32 ± 7.689	26.29 ± 6.057
	Urban	1008	3.68 ± 4.167	34.67 ± 13.730	29.11 ± 7.973	26.00 ± 6.162
	T		10.367	12.326	14.984	0.678
	P		0.001	0.001	0.001	0.411

N, sample size; T, t-test statistic; F, ANOVA statistic; P, significance level. ^*^p < 0.05, ^**^p < 0.01, ^***^p < 0.001.

Based on the recommended cutoff for the MPAI (total score ≥ 41; Leung, 2008; Hamilton et al., 2012), 410 of the 1,422 participants (28.76%) were classified as having a Smartphone addiction. The prevalence among male students was 30.08% (271/901), and among female students, it was 26.68% (139/521). This prevalence rate is consistent with findings from recent cross-sectional studies on Chinese college students, which report rates between 25.3% and 31.2% (Billieux et al., 2015; Kwon et al., 2013), supporting the representativeness of our sample. All participants signed informed consent forms, fully understood the research purpose and procedures, and participated voluntarily. This study was approved by the Ethics Committee of Jishou University (Approval No.: JS-DX-2023-0034). To ensure data quality, the following criteria were used to exclude invalid questionnaires: (1) identical answers throughout the questionnaire or patterned responses, indicating careless completion; (2) incomplete responses.

### 2.2 Research instruments

#### 2.2.1 Mobile phone addiction index

The Mobile Phone Addiction Index (MPAI) developed by Leung (2008) was used to measure college students' Mobile Phone Addiction levels. This scale was chosen for its well-established reliability and validity among Chinese university students (Hamilton et al., 2012). The scale consists of 17 items covering four dimensions: withdrawal symptoms, loss of control, inefficiency, and escape, which effectively capture the core addiction behaviors central to this study. A 5-point Likert scale was employed, ranging from 1 (completely disagree) to 5 (completely agree), with total scores ranging from 17 to 85 points. Higher scores indicate more severe Mobile Phone Addiction. In this study, the Cronbach's α coefficient for the total scale was 0.91, and the Cronbach's α coefficients for the four dimensions were 0.80, 0.84, 0.82, and 0.91, respectively, indicating good internal consistency. We acknowledge that as an older instrument, the MPAI has limitations in the current smartphone era, and future work could benefit from cross-validation with newer scales like the SAS-SV.

#### 2.2.2 Depression symptoms

The Patient Health Questionnaire-9 (PHQ-9) developed by Kroenke et al. (2001) was used to assess depression symptoms among college students. This scale was developed based on the diagnostic criteria for Major Depressive Disorder (MDD) in the Diagnostic and Statistical Manual of Mental Disorders (4th edition) (DSM-IV; American Psychiatric Association, 2022). It includes 9 items corresponding to the 9 core symptom criteria in DSM-IV, using a 4-point rating scale (0 = not at all, 3 = nearly every day). Total scores range from 0 to 27 points, with higher scores indicating more severe depression symptoms. In this study, the Cronbach's α coefficient for this scale was 0.88, indicating good reliability.

#### 2.2.3 Bedtime procrastination

Bedtime procrastination behaviors were assessed using the Bedtime Procrastination Scale (BPS) (Kroese et al., 2014). This scale consists of 9 items using a 5-point Likert scale, ranging from 1 (almost never) to 5 (almost always), with items 2, 3, 7, and 9 reverse-scored. Based on previous research, this scale has a single-factor structure. The mean score of the 9 items was used as the scale score, with scores ranging from 1 to 5 points. Higher scores indicate more severe bedtime procrastination behavior. In this study, the Cronbach's α coefficient for this scale was 0.86, indicating good reliability.

#### 2.2.4 Psychological resilience

The abbreviated version of the Connor-Davidson Resilience Scale (CD-RISC-10) developed by Campbell-Sills and Stein (2007) was used to measure psychological resilience levels among college students. This scale consists of 10 items using a 5-point Likert scale, ranging from 0 (never) to 4 (always), with total scores ranging from 0 to 40 points. Higher scores indicate higher levels of psychological resilience. In this study, the Cronbach's α coefficient for this scale was 0.97, indicating excellent internal consistency.

### 2.3 Quality control

To ensure data quality, the following measures were implemented: (1) Experienced researchers provided standardized training to the physical education teachers who participated in the survey to ensure understanding of the research objectives and questionnaire administration requirements; (2) Questionnaires were administered collectively by class units using standardized instructions to ensure consistency; (3) Questionnaires were completed anonymously to protect participants' privacy and enhance response authenticity; (4) Questionnaires were distributed and collected on-site to improve the response rate; (5) Attention check questions were included to identify and exclude invalid questionnaires with careless responses, obvious response patterns, or significant missing information.

### 2.4 Statistical analysis

Data management and analysis were conducted using IBM SPSS Statistics 26.0 and the PROCESS macro for SPSS (Hayes, 2013). Prior to the main analyses, we performed rigorous data preparation and assumption testing. For missing data, which accounted for only 1.27% of the cases (18/1422), we employed multiple imputation (5 iterations) to minimize potential bias. Outliers were then detected using both Z-scores (|Z| > 3) and Mahalanobis distance (*p* < 0.001); this process identified and led to the exclusion of 23 outliers (1.62%). All main results remained stable in a re-analysis without these cases. Furthermore, we conducted a complete set of assumption tests for our regression models (Kline, 2015). These tests confirmed that all residuals were approximately normally distributed (Shapiro-Wilktest, *p* > 0.05), the assumption of homoscedasticity was supported (Levene's test, *p* > 0.05), and all variance inflation factors (VIF < 2.0) indicated no issues with multicollinearity. First, Harman's single-factor test was used to examine common method bias. Second, descriptive statistics (means, standard deviations) of the main variables were calculated. Third, Pearson correlation analysis was conducted to examine the relationships among Mobile Phone Addiction, depression symptoms, bedtime procrastination, and psychological resilience. Finally, PROCESS Model 4 and Model 15 (Hayes, 2013) were used to test mediation and moderation effects. Model 4 was used to construct a mediation model with Mobile Phone Addiction as the independent variable, depression symptoms as the dependent variable, and bedtime procrastination as the mediator. Building on this, Model 15 was used to incorporate psychological resilience as a moderator to examine its moderating effects on the relationships between Mobile Phone Addiction and depression symptoms, as well as between bedtime procrastination and depression symptoms. All analyses employed bias-corrected bootstrapping with 5,000 resamples to calculate 95% confidence intervals (95% CI), with a significance level set at α = 0.05.

## 3 Results

### 3.1 Common method bias test

Harman's single factor test was used to examine common method bias. The results showed that 12 factors had eigenvalues greater than 1, with the first factor explaining 37.4% of the variance, which is below the critical threshold of 40%. This indicates that there was no serious common method bias in the data of this study (Podsakoff et al., 2003).

### 3.2 Descriptive statistics and correlation analysis

The analysis results showed that depression symptoms were significantly positively correlated with Mobile Phone Addiction (*r* = 0.456, *p* < 0.01) and bedtime procrastination (*r* = 0.308, *p* < 0.01), and significantly negatively correlated with psychological resilience (*r* = −0.331, *p* < 0.01). Mobile Phone Addiction was significantly negatively correlated with psychological resilience (*r* = −0.291, *p* < 0.01) and significantly positively correlated with bedtime procrastination (*r* = 0.396, *p* < 0.01). Psychological resilience was significantly negatively correlated with bedtime procrastination (*r* = −0.284, *p* < 0.01). The scores of each variable and correlation coefficients are shown in Table 2.

**Table 2:** Scores of each variable and correlation analysis.

Variable	Score (M ±SD)	Depression symptoms	Mobile Phone Addiction	Psychological resilience	Bedtime procrastination
Depression symptoms	3.92 ± 4.278	1			
Mobile Phone Addiction	35.50 ± 13.987	0.456^**^	1		
Psychological resilience	28.59 ± 7.930	−0.331^**^	−0.291^**^	1	
Bedtime procrastination	26.08 ± 6.131	0.308^**^	0.396^**^	−0.284^**^	1

χ±s; Mean ± SD; M, Mean; SD, Standard Deviation. ^*^p < 0.05, ^**^p < 0.01, ^***^p < 0.001.

### 3.3 Mediating role of bedtime procrastination

The results indicated that Mobile Phone Addiction was positively correlated with bedtime procrastination; Mobile Phone Addiction was positively correlated with depression symptoms, and this correlation remained significant after including the mediating variable of bedtime procrastination (see Table 3). There was a significant association between Mobile Phone Addiction and depression symptoms level: the total effect was 1.489 (95% CI [1.338, 1.641]); the direct effect was 1.205 (95% CI [1.053, 1.357]), accounting for 80.92% of the total effect, with the 95% CI not containing 0, indicating that the direct effect of Mobile Phone Addiction on depression symptoms level was significant; the indirect effect was 0.284 (95% CI [0.226, 0.347]), accounting for 19.08% of the total effect, with the 95% CI not containing 0, indicating that the indirect effect of bedtime procrastination between Mobile Phone Addiction and depression symptoms level was significant. Bedtime procrastination played a partial mediating role between Mobile Phone Addiction and depression symptoms. See Table 4.

**Table 3:** Test of the mediating model of bedtime procrastination.

Regression equation		Fit indicators			Significance of coefficients			*P*
Outcome variable	Predictor variable	*R*	*R^2^*	*F*	*B*	*SE*	*t*	
Bedtime procrastination	Mobile Phone Addiction	0.308	0.095	148.335	0.441	0.036	12.179	< 0.001
Depression symptoms	Mobile Phone Addiction	0.456	0.208	371.650	1.489	0.773	19.278	< 0.001
Depression symptoms	Mobile Phone Addiction	0.529	0.280	275.576	1.205	0.774	15.563	< 0.001
	Bedtime procrastination				0.645	0.054	11.934	< 0.001

*R*, Correlation; *R*^2^, R-squared (coefficient of determination); *F*, F-statistic; *B*, Unstandardized regression coefficient; *SE*, Standard error; *t*, t-statistic. ^*^p < 0.05, ^**^p < 0.01, ^***^p < 0.001.

**Table 4:** Analysis of the mediating effect of bedtime procrastination.

Effect	β(95%CI)	SE	Effect size (%)
Total effect	1.489(1.338 1.641)	0.077	100.00
Direct effect	1.205(1.053 1.357)	0.077	80.92
Indirect effect	0.284(0.226 0.347)	0.031	19.08

β, Standardized regression coefficient; SE, Standard error; CI, Confidence Interval.

### 3.4 Relationship between Mobile Phone Addiction and depression symptoms

Before testing the moderated mediation model, the linearity assumption was examined. Pearson correlation analysis indicated significant linear correlations among smartphone addiction, bedtime procrastination, psychological resilience, and depression symptoms (all *p* < 0.01). The moderated mediation analysis was conducted using Hayes' PROCESS Model 15, which is grounded in multiple linear regression and assumes linear relationships among continuous variables. This analytical framework and linearity assumption are consistent with standard practices for testing conditional effects in behavioral and psychological research (von Elm et al., 2007). Therefore, the linear relationship assumption in the moderation plots is statistically justified.

The results showed a positive correlation between Mobile Phone Addiction and depression symptoms (β = 0.104, *P* < 0.01), apositive correlation between bedtime procrastination and depression symptoms (β = 0.093, *P* < 0.01), and a negative correlation between psychological resilience and depression symptoms (β = −0.109, *P* < 0.01). The interaction between Mobile Phone Addiction and psychological resilience had a significant effect on depression symptoms (β = −0.002, *P* < 0.01), and the interaction between bedtime procrastination and psychological resilience had a significant effect on depression symptoms (β = −0.004, *P* < 0.01), indicating that psychological resilience significantly moderated the influence of bedtime procrastination on depression symptoms. See Table 5.

**Table 5:** Moderated mediation effect test for the relationship between depression symptoms and Mobile Phone Addiction.

Regression equation		Fit indicators			Significance of coefficients			*P*
Outcome variable	Predictor variable	*R*	*R^2^*	*F*	*B*	*SE*	*t*	
Depression symptoms	Mobile Phone Addiction	0.518	0.269	103.942	0.104	0.008	13.204	< 0.001
	Bedtime procrastination				0.093	0.018	5.165	< 0.001
	Psychological resilience				−0.109	0.013	−8.234	< 0.001
	Mobile Phone Addiction^*^Psychological resilience				−0.002	0.001	−2.441	0.001
	Bedtime procrastination^*^Psychological resilience				−0.004	0.002	−2.137	0.033

*R*, Correlation Coefficient; *R*^2^, coefficient of determination; *F*, F-statistic; *B*, Unstandardized regression coefficient; *SE*, Standard error; *t*, t-statistic. ^**^ *p* < 0.01, ^*^ *p* < 0.05.

**Figure 2:**
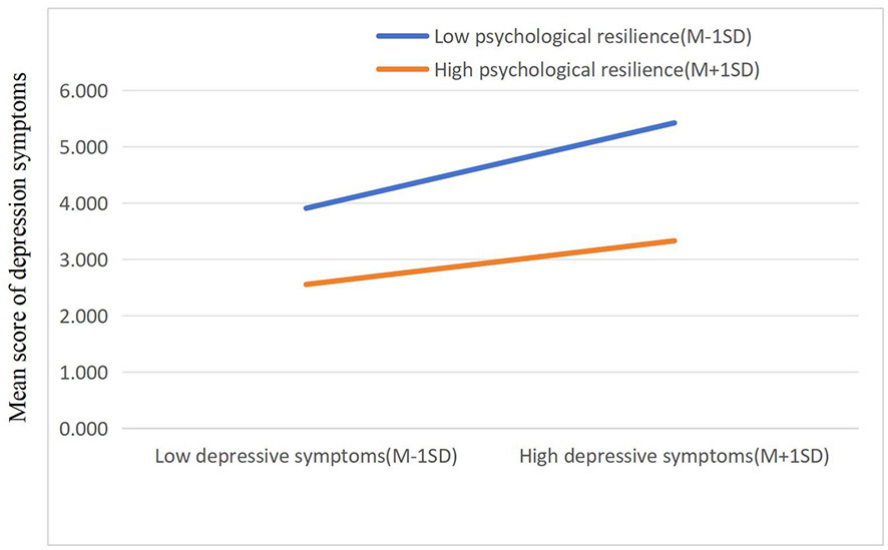
The moderation of psychological resilience between Mobile Phone Addiction and depression symptoms. M, Mean; SD, Standard Deviation; Low vs. high psychological resilience were defined as one standard deviation below the mean (M−1SD) and one standard deviation above the mean (M+1SD), respectively. This categorization is a standard statistical method for probing interaction effects in moderated mediation analysis (Aiken and West, 1991).

Simple slope tests were conducted to further clarify the moderating effects. For the direct pathway (Smartphone addiction → depression symptoms), the positive association was significantly weaker in the high psychological resilience group (β = 0.088, *t* = 7.930, *p* < 0.01) than in the low resilience group (β = 0.120, *t* = 12.917, *p* < 0.01). When psychological resilience was at a high level, the positive correlation between Mobile Phone Addiction and depression symptoms was inhibited, indicating that the higher the level of psychological resilience, the lower the impact of Mobile Phone Addiction on the risk of depression symptoms among college students. This verified that psychological resilience played a moderating role in the direct pathway between Mobile Phone Addiction and depression symptoms (Figure 2). Simple slope plot illustrating the moderating effect of psychological resilience. The figure visually depicts the significant interaction, where the positive association between Mobile Phone Addiction (x-axis) and Depressive Symptoms (y-axis) is weaker for individuals with high psychological resilience compared to those with low resilience. The statistical significance of this slope difference is confirmed by simple slope analysis (*p* < 0.01).

**Figure 3:**
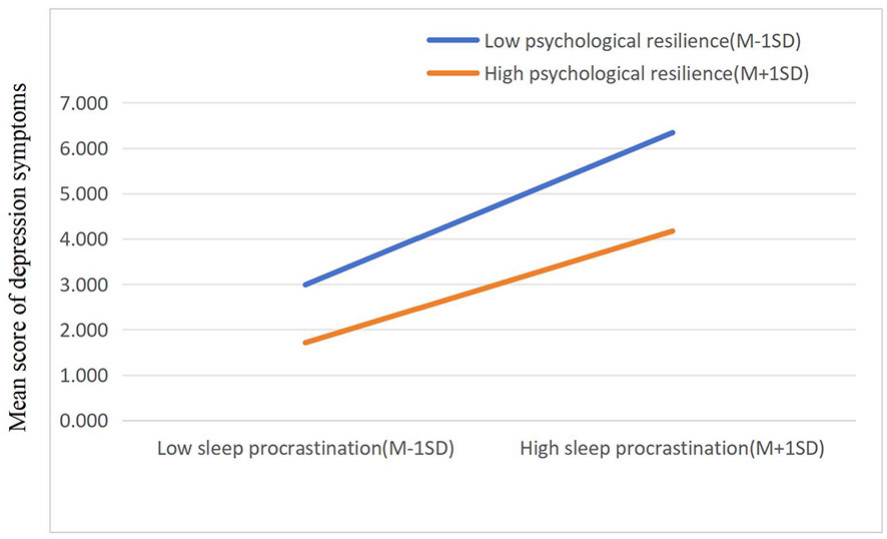
The moderation of psychological resilience between bedtime procrastination and depression symptoms. M, Mean; SD, Standard Deviation; Low vs. high psychological resilience were defined as one standard deviation below the mean (M−1SD) and one standard deviation above the mean (M+1SD), respectively. This categorization is a standard statistical method for probing interaction effects in moderated mediation analysis (Aiken and West, 1991).

Simple slope test showed that the mediation pathway (bedtime procrastination → depression symptoms), the positive association was also significantly weaker in the high resilience group (β = 0.063, *t* = 3.007, *p* < 0.01) than in the low resilience group (β = 0.124, *t* = 4.997, *p* < 0.01). These results confirmed the significant moderating role of psychological resilience. When psychological resilience was at a high level, the positive correlation between bedtime procrastination and depression symptoms was inhibited, indicating that as the level of psychological resilience increased, the impact of bedtime procrastination on the risk of depression symptoms among college students decreased (Figure 3). This further verified that psychological resilience played a moderating role in the latter part of the pathway where bedtime procrastination mediated the relationship between Mobile Phone Addiction and depression symptoms.

## 4 Discussion

This study constructed a moderated mediation model based on the Conservation of Resources (COR) theory to explore the mechanism linking Mobile Phone Addiction to depression symptoms among college students. The findings confirm that is indirectly associated with elevated depressive symptoms through bedtime procrastination, while psychological resilience acts as a critical protective buffer at multiple stages. These results not only elucidate the “how” (mediation) and “when” (moderation) of this relationship but also extend the applicability of COR theory by integrating it with the Social Displacement Hypothesis to explain the unique mental health challenges of the digital age.

### 4.1 The direct impact: Mobile Phone Addiction as a resource depletion spiral

Consistent with Hypothesis 1, Mobile Phone Addiction was found to be a significant positively associated of depression symptoms. Within the COR framework, this direct link represents a “loss spiral.” Individuals addicted to smartphones invest excessive cognitive and emotional resources into virtual interactions (Elhai et al., 2017). However, this digital investment often yields a “net loss” of resources due to the mechanism of social displacement. Excessive screen time displaces face-to-face interactions, which are critical for maintaining high-quality social support—a key buffer against depression (Kushlev and Leitao, 2020). Furthermore, the “always-on” culture creates a state of cognitive fragmentation, where constant task-switching rapidly depletes executive function resources.

When resources are exhausted and cannot be replenished, individuals experience psychological distress, manifesting as depressive symptoms ranging from subthreshold to clinical levels (Hobfoll et al., 2018). Additionally, exposure to idealized images on social media often triggers upward social comparison, threatening self-esteem resources and fostering feelings of inadequacy (Karadag et al., 2015). Unlike the immediate gratification provided by the device, the aftermath of addiction involves social isolation and guilt, further accelerating the depletion of self-esteem resources and precipitating emotional disorders.

### 4.2 The mediating mechanism: bedtime procrastination as failed resource restoration

The study supports Hypothesis 2, identifying bedtime procrastination as a crucial mediator. Sleep is biologically the most essential mechanism for resource restoration. However, our findings suggest that Mobile Phone Addiction disrupts this recovery process, creating a “double jeopardy” effect. This phenomenon is often manifested as “Revenge Bedtime Procrastination,” where students sacrifice sleep to regain a sense of autonomy lost to daytime academic pressures (Kühnel et al., 2018). Mobile Phone Addiction exacerbates this by inducing a “flow” state, diminishing temporal awareness and impairing the self-control resources needed to disengage from screens (Hale and Guan, 2015).

The addiction consumes resources during the day, while the subsequent bedtime procrastination blocks resource replenishment at night (Zhang and Wu, 2020). Crucially, the resulting sleep deprivation acts as a neurobiological stressor. It disrupts the functional connectivity between the amygdala and the medial prefrontal cortex, impairing the brain's “top-down” emotional control mechanisms (Steel, 2007). This pathway illustrates how behavioral dysregulation (procrastination) translates into neurological deficits (sleep loss), which ultimately manifest as psychological pathology (depression).

### 4.3 The moderating role: resilience as a critical resource buffer

Validating Hypotheses 3 and 4, psychological resilience was found to moderate both the direct path (Addiction → Depression) and the second stage of the indirect path (Procrastination → Depression). These findings align with the COR theory's “buffer hypothesis,” which posits that individuals with abundant personal resources can better withstand resource loss. Unlike static traits, resilience here functions as a dynamic affect-regulation process (Troy et al., 2023).

Specifically, for students with high resilience, the correlation between Mobile Phone Addiction and depression was significantly weaker. High-resilience individuals possess superior cognitive flexibility and reappraisal skills, enabling them to reframe the stress of addiction and maintain self-efficacy, thus preventing resource depletion from escalating into despair (Nolen-Hoeksema, 1991). Furthermore, resilience buffered the impact of bedtime procrastination on depression. Even in a state of fatigue caused by sleep delay, resilient individuals can utilize positive coping strategies and maintain higher vagal tone to regulate physiological arousal (Yildirim and Arslan, 2020). This allows them to manage negative affect, effectively “cutting off” the pathway from physiological fatigue to depressive symptoms (Hershner and Chervin, 2014). In contrast, low-resilience individuals, lacking this “safety valve,” are more prone to the catastrophic effects of resource loss, confirming the necessity of resilience-focused interventions.

It is important, however, to interpret the magnitude of these moderation effects with caution. While statistically significant, the interaction coefficients were small, suggesting that psychological resilience explains a modest portion of the variance in the strength of these associations. Following recommendations for evaluating effect sizes (Funder and Ozer, 2019), such small effects may still hold practical significance at a population level. Given the high prevalence of smartphone use and mental health challenges among college students, even a small buffering effect of resilience could translate into a meaningful reduction in depression risk for a large number of individuals. Nevertheless, this finding highlights that psychological resilience is one of several protective factors, and future research should explore other variables that may exert stronger moderating influences.

### 4.4 Limitations and future directions

First, this study adopts a cross-sectional design, which has inherent limitations in causal inference. Specifically, the cross-sectional design can only reveal the associational relationships between Mobile Phone Addiction, bedtime procrastination, psychological resilience, and depression symptoms among college students, but cannot establish directional causal relationships between variables. For example, the existing data cannot rule out the bidirectional association between Mobile Phone Addiction and depression symptoms: while Mobile Phone Addiction may increase the risk of depression symptoms *via* resource depletion and impaired sleep restoration, individuals with higher levels of depression symptoms may also use smartphones excessively as an emotional avoidance and compensatory strategy, which further exacerbates addictive behaviors (dos Santos and de Oliveira, 2022). In addition, the cross-sectional design cannot track the dynamic changes of the variables and the moderated mediation model over time, and cannot explore the long-term impact of Mobile Phone Addiction on the mental health developmental trajectory of college students.

Second, our current models did not account for several potential demographic and contextual confounding variables. Crucial environmental and trait-level factors—such as academic stress, proximity to examination seasons, socioeconomic status, or pre-existing mental health conditions—could plausibly influence both mobile phone use patterns and depressive symptoms, thereby potentially confounding the observed associations (dos Santos and de Oliveira, 2022; Deng et al., 2022). For instance, intense academic stress might simultaneously drive students toward excessive smartphone use as an emotional avoidance strategy while directly contributing to depressive symptomatology. Given the gender imbalance in our sample and the fact that prior research indicates notable gender differences in both mental health vulnerability and digital usage patterns, future studies must strictly control for these covariates or explicitly test them as boundary conditions (moderators) to more accurately isolate the mechanisms within the model.

Future research should address this limitation through more rigorous study designs: (1) Adopt a longitudinal prospective design to track the study cohort for 1–2 academic years, to verify the directional causal relationships between variables and the dynamic stability of the moderated mediation model across different developmental stages of college students; (2) Conduct randomized controlled intervention trials, such as implementing sleep hygiene intervention and psychological resilience training for college students with high levels of Mobile Phone Addiction, to verify the causal pathways proposed in this study through experimental manipulation, and to provide evidence-based intervention solutions for university mental health services and clinical practice.

## 5 Conclusion

This study elucidates the mechanism linking Mobile Phone Addiction to depression symptoms among college students through the lens of Conservation of Resources theory. The results indicate a “resource loss spiral” where Mobile Phone Addiction not only directly predicts depression but also indirectly exacerbates it by triggering bedtime procrastination—a failure of resource restoration. Crucially, psychological resilience serves as a protective buffer, weakening the pathogenic links between addiction, procrastination, and depression. These findings suggest that depression in the digital age is not merely a result of screen time but a consequence of systemic resource depletion. Therefore, intervention strategies should move beyond simple restriction of phone use. Instead, a dual-focus approach is recommended: promoting sleep hygiene to ensure resource recovery, and implementing resilience training programs (e.g., mindfulness or cognitive-behavioral strategies) to build students' internal “resource reservoirs,” thereby enhancing their resistance to digital stress and emotional disorders.

## Data Availability

The raw data supporting the conclusions of this article will be made available by the authors, without undue reservation.
